# Influence of olfactory and visual cover on nest site selection and nest success for grassland‐nesting birds

**DOI:** 10.1002/ece3.3195

**Published:** 2017-07-03

**Authors:** Dillon T. Fogarty, R. Dwayne Elmore, Samuel D. Fuhlendorf, Scott R. Loss

**Affiliations:** ^1^ Department of Natural Resource Ecology and Management Oklahoma State University Stillwater OK USA; ^2^ Present address: University of Nebraska‐Lincoln Lincoln NE USA

**Keywords:** avian nest survival, cover, habitat selection, nest site selection, olfactory concealment, olfactory predators, precipitation, weather

## Abstract

Habitat selection by animals is influenced by and mitigates the effects of predation and environmental extremes. For birds, nest site selection is crucial to offspring production because nests are exposed to extreme weather and predation pressure. Predators that forage using olfaction often dominate nest predator communities; therefore, factors that influence olfactory detection (e.g., airflow and weather variables, including turbulence and moisture) should influence nest site selection and survival. However, few studies have assessed the importance of olfactory cover for habitat selection and survival. We assessed whether ground‐nesting birds select nest sites based on visual and/or olfactory cover. Additionally, we assessed the importance of visual cover and airflow and weather variables associated with olfactory cover in influencing nest survival. In managed grasslands in Oklahoma, USA, we monitored nests of Northern Bobwhite (*Colinus virginianus*), Eastern Meadowlark (*Sturnella magna*), and Grasshopper Sparrow (*Ammodramus savannarum*) during 2015 and 2016. To assess nest site selection, we compared cover variables between nests and random points. To assess factors influencing nest survival, we used visual cover and olfactory‐related measurements (i.e., airflow and weather variables) to model daily nest survival. For nest site selection, nest sites had greater overhead visual cover than random points, but no other significant differences were found. Weather variables hypothesized to influence olfactory detection, specifically precipitation and relative humidity, were the best predictors of and were positively related to daily nest survival. Selection for overhead cover likely contributed to mitigation of thermal extremes and possibly reduced detectability of nests. For daily nest survival, we hypothesize that major nest predators focused on prey other than the monitored species’ nests during high moisture conditions, thus increasing nest survival on these days. Our study highlights how mechanistic approaches to studying cover informs which dimensions are perceived and selected by animals and which dimensions confer fitness‐related benefits.

## 1 INTRODUCTION

Animal habitat selection has major implications for survival, reproductive success, fitness, and population‐level processes, and habitat selection is strongly influenced by both environmental constraints and predation (Caro, 2005; Lima & Dill, 1990; Martin, 1993). Understanding how habitat selection occurs—and how selection influences population parameters—is therefore crucial for effective conservation management. For birds, nest site selection is a key component of habitat selection that influences survival and reproduction (Davis, 2005; Martin, 1993; Martin & Roper, 1988) because eggs and nestlings are sought after by many predators. A substantial body of basic and applied ecological research has addressed relationships among nest site selection, predation, and nest success because these processes have profound implications for predator and prey behavior, life‐history evolution, and avian population management (Clark & Shutler, 1999; Martin, 1992, 1993).

Predators locate prey items, including nests, based on learned suites of sensory cues that can be visual, thermal, aural, and/or olfactory (i.e., search images; Carthey, Bytheway, & Banks, 2011; Nams, 1991; Santisteban, Sieving, & Avery, 2002). Evolutionary theory predicts that, to avoid predation, prey should select habitat that minimizes their signals or sign (e.g., scent, noise, and visual and thermal appearance) used by dominant predators (Van Valen, 1973). Because nest predator communities are often dominated by species that forage primarily using olfaction (hereafter, olfactory predators; Burghardt, 1966; Hughes, Price, & Banks, 2010; Nams, 1997; Slotnick, 2001; Threlfall, Law, & Banks, 2013), selection of nest sites that increase olfactory cover or decrease odor conspicuousness should increase nest survival and potentially reproductive success and fitness. Indeed, research shows that predators are sensitive to the conspicuousness of prey odor cues and use olfactory information to make foraging decisions (Price & Banks, 2016; Threlfall et al., 2013). In general, predator foraging efficiency is relatively high when prey odors are conspicuous and declines as odor becomes less conspicuous (Carthey et al., 2011; Vander Wall, 1998, 2000, 2003). For example, red grouse (*Lagopus lagopus scotica*) with high endoparasite loads produce more odorants and experience higher predation rates than lightly parasitized individuals (Hudson, Dobson & Newborn, 1992).

Weather‐related variables (e.g., wind speed and moisture) can also influence conspicuousness of prey odorants (Borgo & Conover, 2015; Ruzicka & Conover, 2011, 2012; Vander Wall, 1998). As moisture increases, so does the mobility of odorants, and this has been shown to lead to higher foraging efficiency for seed hoarding rodents (Vander Wall, 2003), and in certain cases, higher rates of predation on avian nests (Conover, 2007; Lehman, Rumble, Flake, & Thompson, 2008; Borgo & Conover, 2015; but see Pleasant, Dabbert, & Mitchell, 2003; Moynahan, Lindberg, Rotella, & Thomas, 2007). Additionally, some olfactory predators increase foraging activity at intermediate wind speeds (Ruzicka & Conover, 2011) but appear to have reduced foraging success at high wind speeds (Ruzicka & Conover, 2012).

Airflow characteristics in particular are thought to influence predator detection of airborne odor cues. Specifically, turbulence (i.e., variability in airflow direction and velocity) mixes and homogenizes heat, moisture, and airborne particles (De Visscher, 2013; Stull, 2006), and airborne odor molecules are thought to behave similarly (i.e., with odorants dispersing rapidly, becoming less conspicuous, and thus becoming difficult to detect and track to a source in high‐turbulence conditions) (Conover, 2007). Additionally, updrafts are expected to elevate odor plumes above the detection height of ground‐based predators, thus reducing the ground area over which odor plumes are detectable (Conover, 2007). Turbulence and updraft are both influenced by surface features (e.g., topography, vegetation canopies) (De Visscher, 2013; Stull, 2006) and may be incorporated into prey habitat selection decisions (Conover, 2007; Conover & Borgo, 2009). The few studies addressing potential selection for these factors at nest sites have found they are not selected for (Borgo & Conover, 2016; Conover, Borgo, Dritz, Dinkins, & Dahlgren, 2010); however, the species studied construct open‐cup nests, which are presumably most susceptible to visual‐based predators, thus making visual cover more important than olfactory cover.

Despite the evidence that olfaction and olfactory‐related variables play an important role in avian nesting ecology, the vast majority of research has focused primarily on visual aspects of cover. Here, we conducted an observational study to examine the role of olfactory cover in nest site selection and nest success of grassland‐nesting birds—a guild of conservation concern due to the dramatic loss of grasslands (Hoekstra, Boucher, Ricketts, & Roberts, 2005). Specifically, we (1) assessed whether dome‐nesting birds in grasslands select nest sites based on visual cover and/or airflow characteristics influencing olfactory cover and (2) examined the relative role of visual cover, as well as airflow characteristics and weather conditions associated with olfactory detection of odorants, in influencing nest survival. We hypothesize that ground‐nesting birds in grasslands select nest sites for both visual and olfactory cover due to the prevalence of olfactory predators and the many studies documenting selection for visual cover (e.g., Latif, Heath, & Rotenberry, 2012; Martin, 1992; Weidinger, 2002). In addition, we hypothesize that nest survival is best predicted by factors influencing olfactory cover (e.g., high turbulence, updrafts, moisture, and/or wind speed) because olfactory predators are generally the predominant nest predators in grasslands (see below; Lusk, Smith, Fuhlendorf, & Guthery, 2006; Pietz & Granfors, 2000; Renfrew & Ribic, 2003; Staller, Palmer, Carroll, Thornton, & Sisson, 2005). This study provides novel perspective on the mechanisms behind habitat selection patterns—as well as on the concealment and survival benefits provided by cover—and, therefore, useful insight for effectively managing habitat for prey species of conservation concern.

## 2 MATERIALS AND METHODS

### 2.1 Study system

**Figure 1:**
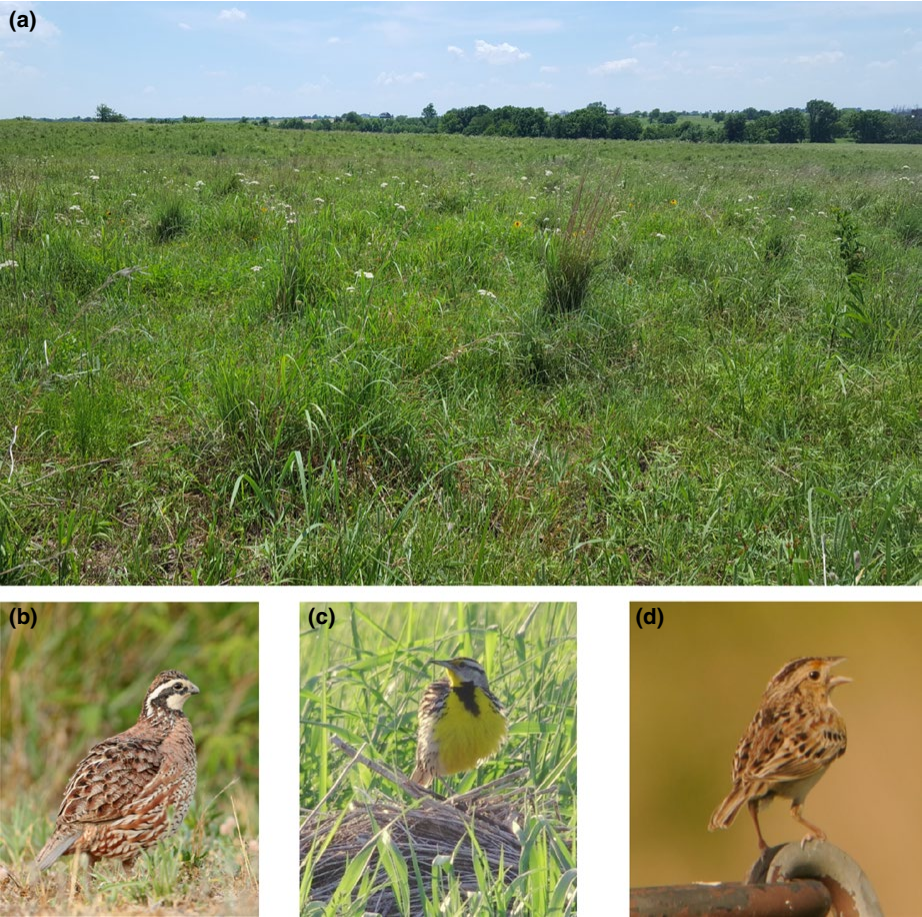
(a) Photograph depicting tallgrass prairie nesting habitat on the McFarlin‐Ingersoll ranch located in Inola, Oklahoma, USA (2016). Photograph of a (b) Northern Bobwhite, (c) Eastern Meadowlark, and (d) Grasshopper Sparrow. Photography courtesy of (a) D. Fogarty, (b) M. Tillett (CC BY 2.0), (c) CheepShot (CC BY 2.0), and (d) A. Reago and C. McClarren (CC BY 2.0)

**Figure 2:**
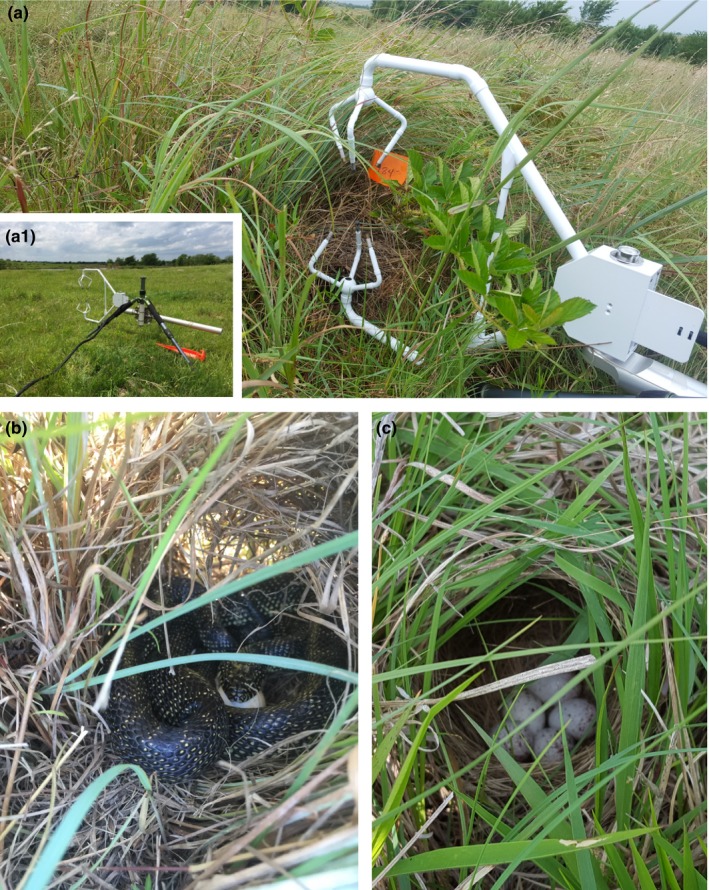
(a.1) Sonic anemometer mounted on a camera tri‐pod (a) recording airflow velocity readings at a Northern Bobwhite nest. (b) A speckled kingsnake depredating a bobwhite nest and (c) a Eastern Meadowlark nest with a full clutch of eggs. All photographs were taken on the McFarlin‐Ingersoll ranch, Inola, Oklahoma, USA, in 2015 and 2016. Photographs are courtesy of (a, a.1, c) D. Fogarty and (b) C. Fitzmorris

Between May and August of 2015 and 2016, we monitored nests of Northern Bobwhite (hereafter bobwhite) (*Colinus virginianus*; order Galliformes and family Odontophoridae), and in 2016, we also monitored nests of Eastern Meadowlark (hereafter meadowlark) (*Sturnella magna*; order Passeriformes and family Icteridae) and Grasshopper Sparrow (*Ammodramus savannarum*; order Passeriformes and family Emberizidae) (Figure 1b–d). These ground‐nesting species construct structurally similar dome‐shaped nests made of dead grasses and forbs placed in or near tussocks of bunchgrasses (Figure 2c). All three species can make multiple nesting attempts each breeding season, with each attempt consisting of a newly constructed nest. Clutch sizes are 12–6 eggs for bobwhite, 3–5 eggs for meadowlark, and 3–6 eggs for grasshopper sparrow. Meadowlark and grasshopper sparrow have altricial young, thus each nesting attempt consists of both incubation and nestling periods. Bobwhite young are precocial and immediately leave the nest after hatching. When approached by a potential threat, each species typically remains on the nest until the threat becomes imminent, at which point they flee, often trying to entice predators away from the nest with a distraction display (see below for information about the predator community).

**Figure 3:**
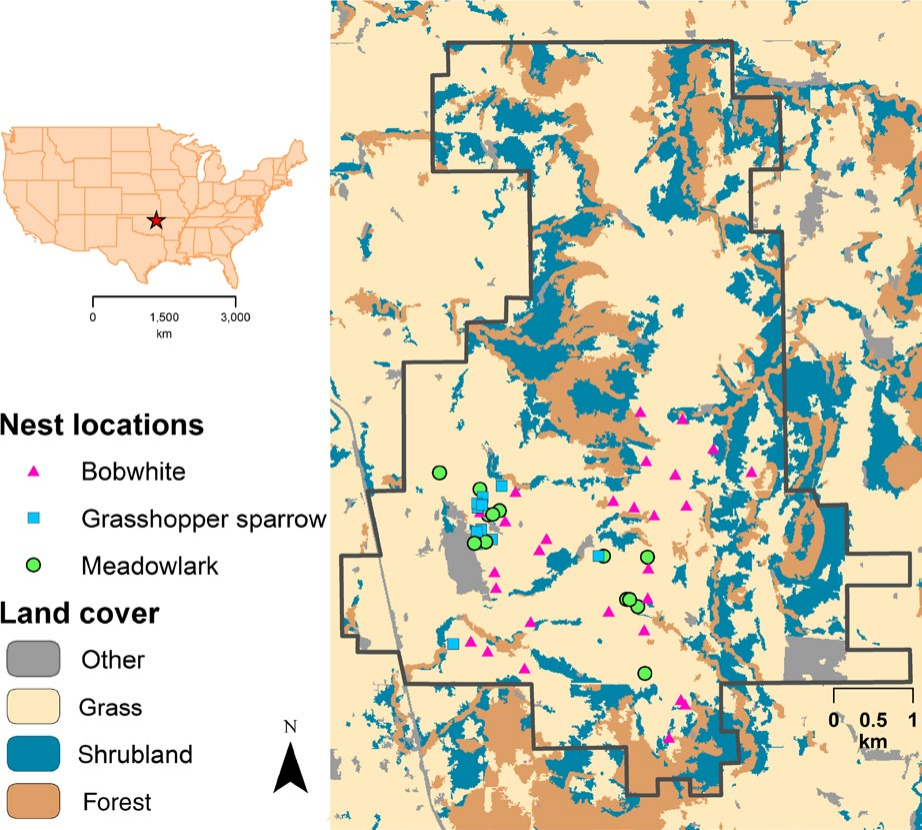
Location of study area in the United States (star), approximate locations of monitored avian nests in 2015 and 2016, and major land cover types of the McFarlin‐Ingersoll ranch, Inola, Oklahoma, USA

The study area was located on the 4,692‐ha McFarlin‐Ingersoll ranch (see below for information about management), 45 km east of Tulsa, Oklahoma, USA (230 m elevation) (latitude: 36.222915; longitude: −95.494537) (Figure 3). Located within the central irregular plains ecoregion, the study area consists largely of tallgrass prairie (~62% of area) (Figure 1a), with patches of forest (~15%) and shrubland (~20%) occurring near creeks and draws, on hillsides, and in low elevation areas. Common grasses included little bluestem (*Schizachyrium scoparium*), switchgrass (*Panicum virgatum*), and big bluestem (*Andropogon gerardi*); common forbs included southern ragweed (*Ambrosia bidentate*), and antelope‐horn milkweed (*Asclepias viridis*); common shrub species included Oklahoma blackberry (*Rubus oklahomus*) and coralberry (*Symphoricarpos orbiculatus*). During the 2015 and 2016 nest monitoring periods, mean daily temperature was 24°C and mean daily maximum temperature was 30°C. Precipitation occurred on 45 of 123 days in 2015 (70 total cm) and 29 of 93 days in 2016 (31 total cm). Mean daily dew point and relative humidity were 19°C (minimum 4°C; maximum 25°C) and 77% (minimum 55%; maximum 98%), respectively (weather information from Oklahoma Mesonet [see below]; Brock et al., 1995; McPherson et al., 2007).

Potential nest predators that we observed within the study area consisted of a suite of mammal, snake, and avian species. Mammals observed included coyote (*Canis latrans*), Virginia opossum (*Didelphis virginiana*), striped skunk (*Mephitis mephitis*), northern raccoon (*Procyon lotor*), nine‐banded armadillo (*Dasypus novemcinctus*), white‐tailed deer (*Odocoileus virginianus*), eastern wood rat (*Neotoma floridana*), and other unidentified small mammals (Muridae family). The most common snake species observed in the vicinity of nests was the speckled kingsnake (*Lampropeltis getula holbrooki*) (Figure 2b). Previous research indicates that mammalian and snake species have highly developed olfactory systems, which are relied upon while foraging (Burghardt, 1966; Conover, 2007; Hughes et al., 2010; Nams, 1997; Shivik & Clark, 1997; Slotnick, 2001; Threlfall et al., 2013). Potential avian nest predators that we observed included American crow (*Corvus brachyrhychos*), blue jay (*Cyanocitta cristata*), red‐tailed hawk (*Buteo jamaicensis*), and turkey vulture (*Cathartes aura*). Avian predators, with the exception of turkey vultures, are thought to rely on visual systems during foraging (Dwernychuk & Boag, 1972; Santisteban et al., 2002).

Studies that have identified nest predators for our study species have shown the dominant predators to be snakes, northern raccoon, Virginia opossum, striped skunk, and small mammals (Hernandez, Rollins, & Cantu, 1997; Pietz & Granfors, 2000; Renfrew & Ribic, 2003). Further, these nest predators have collectively been shown to depredate nests at all times of day and night (Pietz & Granfors, 2000; Staller et al., 2005). The scarcity of predation events by avian predators may be due to the difficulty of visually locating nest contents concealed by a dome‐like structure.

The primary land use on our study area was cow–calf (*Bos tauras*) production, and during the 2015–2016 study period, there was an average stocking rate of 3.1 hectares per animal unit (ha per AU). From October to April, the study area was also used for occasional competition bird–dog trials during which roughly 1,200 captive‐reared bobwhite were released annually; however, no trials were conducted while monitored nests were active. To distinguish these captive‐reared birds from wild‐hatched bobwhite, leg bands with unique numeric codes were attached to all released bobwhite in 2015 and 2016. However, some wild bobwhite presumably bred with captive‐raised birds prior to the onset of our study (DeVos & Speake, 1995); therefore, an unknown proportion of the bobwhite nests we monitored may have represented birds with a mix of wild and captive‐reared provenance.

### 2.2 Data collection

#### 2.2.1 Nest location and monitoring

Between 1 February and 15 July 2015 and 2016, we captured bobwhite with funnel traps (Stoddard, 1931), and to all wild (i.e., unbanded) bobwhite, we attached a uniquely numbered leg band and 6 g VHF radio‐collar (Advanced Telemetry Systems, Isanti, Minnesota, USA); radio‐collars are very commonly used in bobwhite nesting research (e.g., Carroll, Davis, Elmore, & Fuhlendorf, 2015; Lusk et al., 2006) and were <4% of bobwhite body mass. We monitored bobwhite for nesting activity with radio‐telemetry on a daily basis between April and July. All bobwhite nests were found by searching areas where bobwhite were repeatedly observed via telemetry at the same location. To locate meadowlark and grasshopper sparrow nests in 2016, we selected areas with appropriate vegetation structure (grassland) for these species (Fisher & Davis, 2010; Hovick, Elmore, Fuhlendorf, Engle, & Hamilton, 2015). Two or three observers simultaneously walked parallel ~250 m transects spaced 1 m apart from 800 to 1,200. When birds were flushed, we visually searched the general area for a nest. All nest locations were marked with a handheld GPS unit, and nests were monitored every 1–3 days until completion. Nests were considered successful if ≥1 young successfully left the nest and failed if no young successfully left the nest. We confirmed nests as successful by checking the nest around the time of the estimated completion date and observing young or parental agitation and/or defense behaviors near the nest. We were unable to age nests of grasshopper sparrow and meadowlark that were found with a full clutch of eggs and were depredated before nestlings hatched, the point at which completion date would have been estimated. All animal capture and handling procedures were approved by The Institutional Animal Care and Use Committee at Oklahoma State University (IACUC; Protocol No. AG‐14‐25).

#### 2.2.2 Collection of habitat variables influencing olfactory and visual concealment

Measurement of all vegetation and airflow variables was conducted at nest sites and random points between 1,000 and 1,700 from 16 April to 21 August 2015 and 2016 on days when ambient wind speeds were between 7 and 24 km/hr. This range of wind speeds is representative of average conditions experienced in the study area and contains the range of wind speeds thought to correspond to favorable conditions for olfactory detection (Brock et al., 1995; McPherson et al., 2007; Ruzicka & Conover, 2011). To avoid disturbing active nests, we measured variables at all nests immediately after completion, and random points were measured throughout the nesting season. For random sampling we used geospatial modeling environment software (Beyer, 2012) to create 40 randomly located clusters within grassland land cover, each consisting of three sampling points separated by ≥50 m and with all clusters separated by ≥100 m (Fogarty, 2017 *in press*). Of these 40 clusters, we sampled at 110 points; the remaining 10 points were found to not be characteristic of grassland land cover and were therefore not sampled (Fogarty, 2017 *in press*). When locating random points, we used a GPS unit to locate the point and dropped a pin designating the sample point once the GPS indicated a distance of zero meters from the sample point.

To characterize olfactory cover at all nest sites and random points, we used a sonic anemometer (CSAT3, Campbell Scientific, Utah, USA) to measure airflow velocity in three dimensions, with measurements taken every second for 30 min at a height of 25 cm above ground. We used a camera tripod to mount and level the anemometer (Figure 2a.1), and to prevent the structure of the anemometer and tripod from influencing measurements, we faced the anemometer into the direction of the wind. Airflow measurements corresponded to a *u*,*v*,*w* coordinate system where the *u*‐axis was parallel to a horizontal plane aligned with the direction of the wind, the *v*‐axis was parallel to a horizontal plane and perpendicular to the *u*‐axis, and the *w*‐axis was vertical (velocity resolution in the *u*_*u*_ and *u*_*v*_ planes was 1 mm/s, and velocity resolution in the *u*_*w*_ plane was 0.5 mm/s). For each point, velocity (*U*; m/s) was calculated for every second (i.e., 1,800 total measurements across the 30‐min period) as the square root of *u* ^2^ + *v* ^2^. A single estimate of turbulence (*T*) was then calculated for each point as the standard deviation of all velocity measurements. Because *T* is positively correlated to *U*, we used turbulence intensity (TI; calculated as *T*/*U*) in our analyses. TI is a dimensionless measure of turbulence and represents an index of lateral odor plume dispersal (Conover, 2007). All of these airflow calculations are standardly used in boundary layer meteorology (Stull, 1988) and were also used by Conover et al. (2010) in a previous habitat selection study. To characterize the tendency for air to rise or fall relative to distance from an odor source, we first calculated average velocity on the *w*‐axis (*W*), and then divided by *U* to calculate airflow slope (WU). Airflow slope indirectly captures updraft by providing an index for the horizontal distance over which an odor plume remains within a range of height detectable to ground‐based predators.

At each point, we also quantified visual cover variables, including grass height, horizontal cover, and overhead cover. Grass height has frequently been shown to be selected for by birds and has previously been related to visual, olfactory, and thermal aspects of cover (Conover, 2007; Fogarty, 2017 *in press*; Hovick, Elmore, Allred, Fuhlendorf, & Dahlgren, 2014). Grass height was recorded at the tallest blade/stem of grass in a 1 m^2^ plot centered at each sample point (Davis, 2005; Hovick et al., 2014). To measure horizontal cover, we visually estimated percent visual obstruction starting at ground level in 20% increments (e.g., 0%–20%, 21%–40%, etc.) for each of four 10‐cm‐tall segments on a 2.5‐cm width cover pole (similar to Griffith & Youtie, 1988). Observations were taken from a height of 1 m and a horizontal distance of 4 m in each of 4 cardinal directions, and all obstruction estimates for each point were averaged to generate an index of horizontal cover within 40 cm of ground‐level. To measure overhead cover, we used the angle of obstruction method (AOB) (Kopp, Guthery, Forrester, & Cohen, 1998). For AOB, a pole and digital level are used to record the angle in the vertical plane (0–90°, starting at 90° straight above the point) at which a direct line of sight from 1.5 m to ground level is first obstructed (90° indicates complete obstruction). This measurement was repeated at each cardinal and sub‐cardinal direction (*n* =* *8) and averaged to provide an index of cover from above, a measurement relevant to microclimate (i.e., overhead cover relates to shade) and detection by avian predators (Carroll, Davis, Fuhlendorf, & Elmore, 2016; Kopp et al., 1998).

#### 2.2.3 Collection of weather variables influencing olfactory concealment

For each day that vegetation and airflow measurements were conducted, weather data were accessed from the Oklahoma Mesonet database from a weather station in Inola, Oklahoma, 7.5 km southeast of the study area (Brock et al., 1995; McPherson et al., 2007). All weather variables accessed (hereafter weather olfactory variables) have previously been associated with altering the detectability of odorants. Variables compiled included several measurements of moisture: soil moisture for the top 5 cm (hereafter soil moisture), percent relative air humidity (hereafter humidity), and daily precipitation (hereafter precipitation)—which was also used to create a variable reflecting a 1‐day lag effect of precipitation (i.e., amount of precipitation the previous day, hereafter previous‐day precipitation). It is well established that moisture influences olfactory detection of prey items (e.g., nests, carcasses, cached seeds), which may be due to the increased mobility of odorants during high moisture conditions (Conover, 2007; Vander Wall, 1998, 2000, 2003) contributing to higher foraging success for olfactory predators (Ruzicka & Conover, 2012; Vander Wall, 1998). Additionally, some studies have shown a lag effect of precipitation one day after a rain event (Moynahan et al., 2007; Webb et al., 2012). In addition to moisture variables, we also extracted a single wind speed variable: wind speed at 2 m above ground level. High winds disperse odor plumes, and the rate of odor dispersal has been shown to influence depredation rates (Ruzicka & Conover, 2012; Webb et al., 2012). When the time between nest monitoring visits (hereafter exposure period) was >1 day, we averaged all weather variables over the exposure period.

### 2.3 Statistical analyses

Across both years, we found 32 bobwhite nests, and in 2016, we found 11 grasshopper sparrow and 14 meadowlark nests (57 total nests). Of these, we measured habitat characteristics at 50 nests (26 bobwhite, 13 meadowlark, and 11 grasshopper sparrow nests). At the remaining seven nests, vegetative structure was severely altered by livestock before data could be collected and was therefore not representative of conditions during the nesting period. For the nest survival analysis, we also removed nests that did not survive through at least one exposure period, a requirement of the logistic exposure modeling approach (Shaffer, 2004). We also removed abandoned nests because they were not relevant to an evaluation of nest predation as we could not confirm whether abandonment was predator‐induced. After implementing these steps, 44 nests remained for the nest survival analysis (21 bobwhite, 12 meadowlark, and 11 grasshopper sparrow nests). For both nest site selection and nest survival analyses, we pooled nests for all species to allow general assessment of olfactory cover hypotheses for ground‐nesting birds in grasslands and also because sample size constraints limited separate analyses for each species.

All analyses were conducted in R version 3.2.2 (R Core Team, 2015). For both nest site selection and daily nest survival analyses, we used a mixed effects modeling framework. We treated species as a random effect, assuming varying intercepts and fixed slopes (i.e., a random‐intercepts model), to account for potential dissimilarities among species (e.g., in the amount and type of odor produced). To assess whether birds select nest sites for olfactory and/or visual cover, we compared all vegetation and airflow variables between nest sites (*n *=* *50) and random grassland sites (*n *=* *110) using linear mixed models (LMMs; lmer function in package lme4). For each vegetation or airflow variable—including grass height, overhead cover, horizontal cover, turbulence intensity, and airflow slope—we defined a model with the vegetation or airflow variable as the response variable and point type (nest or random un‐used) as a fixed‐effect. We assessed significance of vegetation and airflow variables using a likelihood ratio test comparing each above model to a null model with the same random‐effect structure (significance determined at α = 0.05).

To assess the relative importance of visual cover, as well as airflow and weather conditions associated with olfactory cover, in predicting daily nest survival probability, we used generalized linear mixed models (GLMMs; glmer function in package lme4) with a binomial error distribution and the logistic exposure link function (Shaffer, 2004). This nest survival modeling approach accommodates temporally varying predictor variables (e.g., precipitation), and the link function takes into account the length of the exposure period when calculating daily survival probabilities. For each category of potential predictor variables—visual cover, airflow olfactory cover, and weather olfactory—we created candidate models of univariate and additive models based on the above‐described support from the literature. All models were compared, along with a null model with the same random‐effects structure, using Akaike's information criterion corrected for small sample sizes (AIC_*c*_) (Burnham & Anderson, 2002). Assessment of model support was based on ΔAIC_*c*_ values (ΔAIC_*c*_ values 0–2 indicating strong support), AIC_*c*_ weights, and model support relative to the null model.

## 3 RESULTS

### 3.1 Nest site selection

**Figure 4:**
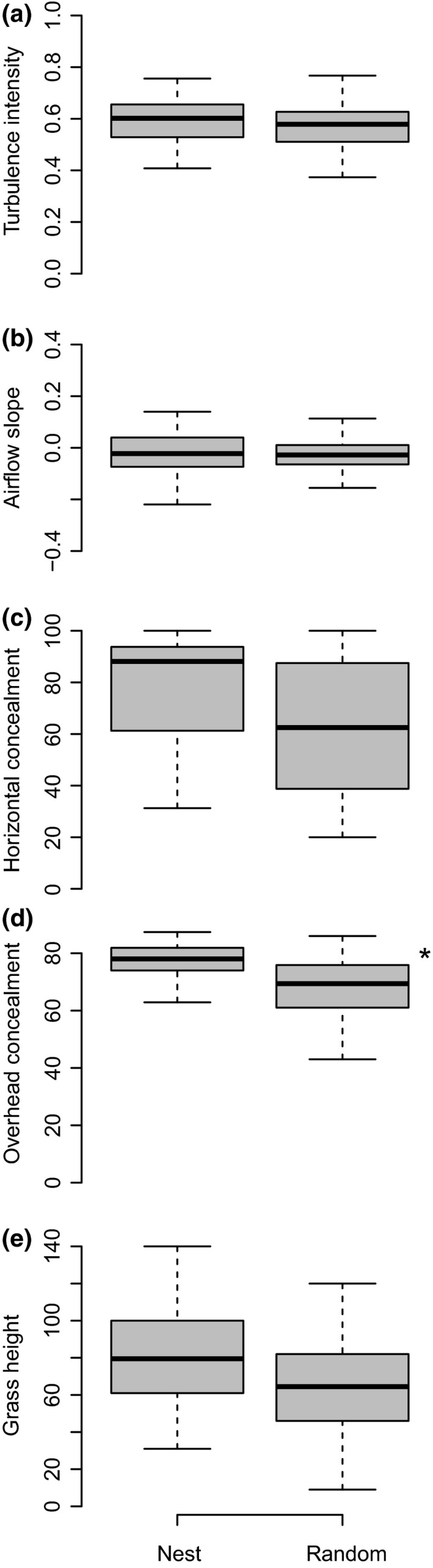
Mean and quartiles for (a) turbulence intensity, (b) airflow slope, (c) horizontal cover, (d) overhead cover, and (e) grass height at Northern Bobwhite, Eastern Meadowlark, and Grasshopper Sparrow nest sites and random grassland sites measured in 2015 and 2016 on the McFarlin‐Ingersoll ranch, Inola, Oklahoma, USA. *indicates a significant difference between nest sites and random sites

Likelihood ratio tests relative to the null model indicated that turbulence intensity, airflow slope, horizontal cover, and grass height were not significantly different between nest sites and random points (χ^2^ ≤ 2.66, *df* = 1, *p *≥* *.10) (see Figure 4 and Table Table S1 for β ± *SE* and *p*‐values). Overhead cover (χ^2^ = 9.13, *df* = 1, *p *<* *.01) was significantly greater at nest sites (β ± *SE* = 77.26 ± 1.45) compared to random sites (β ± *SE* = 67.00 ± 1.79).

### 3.2 Daily nest survival modeling

A total of 44 nests (21 bobwhite, 12 meadowlark, and 11 grasshopper sparrow nests) were used to model daily nest survival, and of these, 10 nests (two bobwhite, six meadowlark, and two grasshopper sparrow nest) were successful. Because we removed abandoned nests (i.e., included nests were either successful or depredated), survival rates directly reflect probability of surviving depredation. Average daily survival rate estimated from the null model (all following daily survival rate estimates include ± *SE*) was 0.916 ± 0.001.

**Figure 5:**
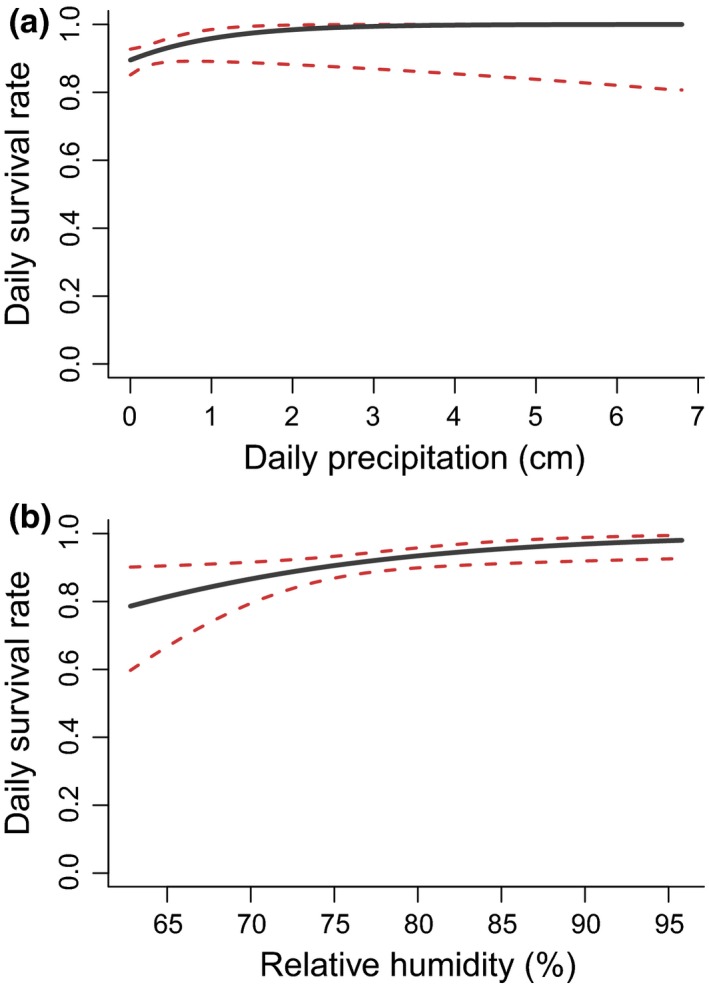
Modeled relationship from generalized linear mixed models (GLMMs) for daily nest survival probability and (a) daily precipitation (cm) and (b) daily relative humidity (%) for Northern Bobwhite, Eastern Meadowlark, and Grasshopper Sparrow nests during 2015 and 2016 on the McFarlin‐Ingersoll ranch, Inola, Oklahoma, USA

To assess the relative importance of visual cover, as well as airflow and weather variables associated with olfactory cover, in influencing daily nest survival probability, we evaluated 18 candidate models (one null model, three visual cover models, three airflow olfactory cover models, and 11 weather olfactory models; Table 1). Of these, four weather olfactory models, but no airflow olfactory or visual cover models, were strongly supported (ΔAIC_*c*_ < 2), indicating that weather olfactory variables most strongly influenced daily survival rate (Table 1). The top model (ΔAIC_*c*_ = 0.0, ω*i* = 0.26) contained precipitation; this variable was positively associated with daily nest survival (β = 1.001 ± 0.576) (Figure 5a), and the model indicated a 0.895 ± 0.020 chance of nest survival on days with no precipitation compared to a 0.999 ± 0.016 chance of nest survival on days with 5 cm of precipitation. The second best model (ΔAIC_*c*_ = 0.9, ω*i* = 0.16) contained humidity, and this variable was also positively associated with nest survival (β = 0.079 ± 0.033) (Figure 5b). The third best model (ΔAIC_*c*_ = 1.2, ω*i* = 0.14) included both precipitation and previous‐day precipitation (β = 0.221 ± 0.265). The fourth best model (ΔAIC_*c*_ = 2.0, ω*i* = 0.09) included both humidity and wind speed (β = 0.033 ± 0.035). However, the standard errors of the β coefficient for wind speed and previous‐day precipitation overlapped zero, indicating a weak effect size of these variables, and these variables also appear to be “uninformative” based on ΔAIC_*c*_ values falling within 2Δ_*i*_ units from the simpler nested models (Arnold, 2010).

**Table 1:** Model selection results for analysis of the influence of visual cover, as well as airflow and weather variables associated with olfactory cover (respectively, referred to as “visual,” “airflow,” and “weather” model type below), on daily nest survival of ground‐nesting birds on the McFarlin‐Ingersoll ranch, Inola, Oklahoma, USA (2015 and 2016). Boldface indicates strongly supported models that do not contain uninformative variables (Arnold, 2010)

Model type	Model	*K* a	ΔAIC_*c*_ b	ω*i* c
Weather	**Precipitation**	3	0.0	0.26
Weather	**Humidity**	3	0.9	0.16
Weather	Precipitation + previous‐day precipitation	4	1.2	0.14
Weather	Humidity + wind speed	4	2.0	0.09
Weather	Precipitation + wind speed	4	2.1	0.09
Weather	Precipitation + previous‐day precipitation + wind speed	5	3.3	0.05
Visual	Vertical cover	3	3.5	0.05
Visual	Overhead cover + horizontal concealment	4	3.9	0.04
Weather	Previous‐day precipitation	3	4.5	0.03
Null	Null	2	4.9	0.02
Visual	Turbulence intensity	3	5.3	0.02
Weather	Previous‐day precipitation + wind speed	4	6.5	0.01
Visual	Horizontal cover	3	6.7	0.01
Weather	Wind speed	3	6.7	
Airflow	Airflow slope	3	6.8	0.01
Airflow	Turbulence intensity + airflow slope	4	6.9	0.01
Weather	Soil moisture	3	6.9	0.01
Weather	Soil moisture + wind speed	4	8.8	0.00

a Number of parameters in the model.

b Difference in AIC_*c*_ value between model and the most strongly supported model.

c AIC_*c*_ Weight ‐ relative strength of support for model.

## 4 DISCUSSION

We found that ground‐nesting birds selected nest sites for overhead visual cover, but there was no clear evidence of selection for turbulence intensity or airflow slope, variables associated with olfactory cover. As described in detail below, overhead cover could provide multiple benefits to nesting birds, including both visual and thermal cover. We also found that weather olfactory variables related to moisture, specifically precipitation and relative humidity, had the greatest influence on and were both positively related to nest survival. Although turbulence intensity and airflow slope did not predict nest survival, precipitation and humidity could influence olfactory detection of nest sites by predators.

### 4.1 Nest site selection

Contrary to our hypothesis that ground‐nesting birds would select nest sites for factors influencing both visual and olfactory cover, we found that only overhead cover was selected for among the variables we measured. We measured overhead cover to provide an index of nest cover from visual‐hunting avian predators and stressful thermal conditions. Below we weigh the evidence for both of these factors that may have driven selection for overhead cover.

Overhead cover and other habitat characteristics expected to influence nest detection by avian predators (e.g., proximity to perch locations) are commonly documented drivers of nest site selection and have been shown to influence nest survival for open‐cup nesting birds (Clark & Shutler, 1999; Dinkins, Conover, Kirol, Beck, & Frey, 2016; Dwernychuk & Boag, 1972; Erikstad, Blom, & Myrberget, 1982). However, selection for overhead cover has not been frequently documented for species that build dome‐shaped nests, including the three bird species in our study (but see Carroll et al., 2015; Townsend et al., 2001). Additionally, studies using cameras to identify nest predator communities for the species in our study have found little if any depredation by avian predators (see predator information in Section 2; Lusk et al., 2006; Pietz & Granfors, 2000; Renfrew & Ribic, 2003; Staller et al., 2005). Therefore, we expect that selection for overhead cover in our study is unlikely to reflect an antipredator behavior.

Although overhead cover could in some cases prevent visual detection of nests by mammalian predators, selection for overhead cover in our study is perhaps more likely to be driven by stressful thermal conditions. Previous studies in the region indicate that increasing overhead cover is associated with cooler microclimates (Carroll et al., 2016; Hovick et al., 2014) and that cooler conditions can be selected for and influence nest survival for ground‐nesting birds (Carroll et al., 2015; Grisham, Godar, Boal, & Haukos, 2016; Hovick et al., 2014). Selection for relatively cooler microclimates allows individuals and their nests to avoid lethal summer temperatures, and this strategy of avoiding extreme heat may have been important in our study area as daily temperatures exceeded 30°C on 91 days during the 2015 and 2016 nesting seasons. Although high temperatures may have plausibly driven selection for high levels of overhead cover due to the cooler conditions it provides, we are unable to definitively confirm this mechanism due to the observational nature of our study. Additionally, our study only quantified aspects of habitat related to cover, and there could have been other habitat variables (e.g., proximity to feeding areas) contributing to nest site selection. However, based on our observations and previous thermal ecology research (Carroll et al., 2015; Carroll et al., 2015b; Hovick et al., 2014), we suggest future research should evaluate the relative influence of thermal cover and other factors in influencing nest site selection.

Although selection for thermal cover has been shown to be important in subtropical grassland ecosystems such as our study area (Carroll et al., 2015; Tanner et al., 2017), there is no evidence that a tradeoff exists between thermal cover and other types of cover (e.g., visual and olfactory). Indeed, vegetation could simultaneously provide multiple mechanisms of protection. For example, tall vegetation has variously been associated with cooler temperatures (Carroll et al., 2016; Hovick et al., 2014), high levels of visual cover (Ganguli et al., 2000), and high levels of turbulence intensity (Fogarty, 2017 *in press*), the latter of which is positively correlated with olfactory cover. Therefore, in many cases, these different dimensions of cover are likely to be positively correlated. Animals likely select and/or benefit from multiple dimensions of cover simultaneously, and additional research is needed to identify how various aspects of habitat cover interactively contribute to fitness under various environmental conditions.

We found that turbulence intensity was not significantly different between nest sites and random points. However, given the observed *p*‐value of 0.10 for this comparison and our limited sample size of 50 nests, we argue that further research is warranted to address whether turbulence intensity is selected for at nest sites. There are several reasons why we expected ground‐nesting birds in grasslands to select high‐turbulence areas for the olfactory cover they provide, including: (1) high levels of turbulence intensity have been shown to decrease the probability that olfactory predators detect a simulated prey item in the same grasslands used for the current study (Fogarty, 2017 *in press*), (2) the only other study evaluating selection for turbulence intensity at nest sites also found a nearly significant higher level of turbulence intensity at nest sites compared to random points despite a limited sample size of nests (Conover et al., 2010) and (3) turbulence, a variable we found to be inversely correlated with turbulence intensity in grasslands (Fig. S1), was previously found to be lower (i.e., ostensibly, turbulence intensity was higher) at successful nests compared to unsuccessful nests for ground‐nesting duck species in grasslands (Borgo & Conover, 2016).

### 4.2 Nest survival

Average daily precipitation and relative humidity during exposure periods were the best predictors of daily nest survival. Contrary to the hypothesis that moisture decreases daily nest survival (Conover, 2007; Roberts, Coffey, & Porter, 1995), we found that precipitation and relative humidity were both positively associated with daily nest survival. That is, nests were more likely to survive on days when it rained or when relative humidity was high (Figure 5). Previous research indicates that the effect of daily precipitation on nest survival is likely context‐dependent, with some studies finding nests more likely to survive on days with precipitation (Conrey, Skagen, Yackel Adams, & Panjabi, 2016; Moynahan et al., 2007; Pleasant et al., 2003; Rader, Brennan, Hernández, Silvy, & Wu, 2007) and others finding survival to be less likely on days with precipitation (Dinkins et al., 2016; Dinsmore, White, & Knopf, 2002; Lehman et al., 2008; Webb et al., 2012). During periods of high moisture (e.g., precipitation or high humidity), water molecules are thought to displace odorants from surface binding sites (e.g., vegetation at bed sites, and eggs, feathers and fur of prey) and thus increase the conspicuousness of odor cues and predator foraging efficiency (i.e., the moisture‐facilitated depredation hypothesis; Roberts et al., 1995; Conover, 2007).

Although some studies have provided empirical support for the moisture‐facilitated depredation hypothesis (Borgo & Conover, 2015; Ruzicka & Conover, 2012), explanations for a positive effect of precipitation on nest success are less certain. One explanation is provided by Moynahan et al. (2007), who documented a positive effect of precipitation on daily nest survival despite depredation rates increasing the day following precipitation events. They hypothesized that parental nest attendance was high and predator activity was low during precipitation events and that the opposite activity patterns occurred on days following precipitation. Despite this explanation, there is a lack of evidence that olfactory predators reduce foraging activity during precipitation events or during times with high humidity (Cresswell & Harris, 1988; Ruzicka & Conover, 2011; Vickery & Bider, 1981), and moreover, we found no support for the effect of previous‐day precipitation in our nest survival analysis. An alternative explanation is that, during a precipitation event prey odorants are released from many prey sources, and olfactory predators focus their attention primarily on the most beneficial (high benefit to cost ratio) prey items, which may or may not include a particular bird species’ nests depending on the predator and prey community. For instance, in systems where a particular bird species’ nests are not a highly beneficial prey source compared to others (e.g., small mammals), nest success for that species would be expected to increase on days with precipitation (as shown by this study), whereas nest success would decrease on days with precipitation in systems where other types of prey are—relative to avian nests—less beneficial. Thus, the effect of moisture on daily nest survival is likely context‐specific. This explanation could help explain why previous research has documented conflicting patterns with regard to the influence of moisture on daily nest survival, and further this explanation does not contradict findings from previous foraging studies assessing the role of olfaction (e.g., Ruzicka & Conover, 2011, 2012; Vander Wall, 1998). Regardless of the mechanism for the positive association between moisture and nest survival, our findings are broadly consistent with other studies that indicate weather can have large impacts on population vital rates (Conrey et al., 2016; Grisham et al., 2016; Morrison & Bolger, 2002).

## 5 CONCLUSIONS

Predation and environmental constraints broadly influence animal habitat selection, survival, and reproductive output (Caro, 2005; Parmesan, Root, & Willig, 2000; Tanner et al., 2017). Our results further illustrate how vegetation and weather variables associated with olfactory cover influence nest site selection and survival, respectively, for a suite of ground‐nesting birds in grasslands. The pattern of nest site selection documented here, specifically selection for high levels of overhead cover, may reflect a strategy used to cope with extreme heat, as suggested by previous studies illustrating that cover can be selected to mitigate thermal extremes (Carroll et al., 2015, 2016; Hovick et al., 2014; Tanner et al., 2017).

While habitat selection can help mitigate the effect of weather extremes, these phenomena cannot be entirely avoided by animals, and extremes such as prolonged drought, intense rainfall events, and intense heat, can strongly influence animal behavior, reproduction, and population dynamics (Albright et al., 2010; Grisham et al., 2016; Mörschel & Klein, 1997; Parmesan et al., 2000). In support of the importance of weather, we found that precipitation and relative humidity had the greatest influence on and were both positively related to nest success. We hypothesize that the influence of precipitation and high humidity on nest survival is context‐specific, capable of increasing or decreasing nest survival depending on the predator and prey community. However, further research is needed to assess predator foraging activity and nest success during high moisture conditions. Nonetheless, because weather can have large impacts on animal populations and community interactions, it is important to understand the mechanism(s) by which animals are impacted by weather and the strategies animals use to mitigate weather's adverse effects. Further research that takes a mechanistic or functional approach to studying organisms and their habitat (see also Van Dyck, 2012) will be necessary for effective conservation given the challenges posed by human‐induced global change (Madliger, 2012; Robertson, Rehage, & Sih, 2013).

## CONFLICT OF INTEREST

None declared.
