# Supplementary material for: Influence of olfactory and visual cover on nest site selection and nest success for grassland‐nesting birds
Source: Ecol Evol. 2017 Jul 3;7(16):6247–58. doi: 10.1002/ece3.3195 (PMC5574794; doi:10.1002/ece3.3195)
Supplement: Supplementary file 1 [file ECE3-7-6247-s001.docx]

**Supporting Information**

**Table S1.** Parameter estimates from linear mixed models and results of likelihood ratio tests for analysis of nest site selection by ground-nesting birds in 2015 and 2016 on the McFarlin-Ingersoll ranch, Inola, Oklahoma, USA.

| Characteristic | | Nest | Random | χ^2^ | *P*-value |
| --- | --- | --- | --- | --- | --- |
|  |  | β ± SE | β ± SE |  |  |
| Olfactory | |  |  |  |  |
|  | Turbulence intensity | 0.59 ± 0.01 | 0.57 ± 0.01 | 2.66 | 0.10 |
|  | Airflow slope | -0.02 ± 0.02 | -0.02 ± 0.02 | 0.15 | 0.70 |
| Visual | |  |  |  |  |
|  | Horizontal concealment | 74.00 ± 11.17 | 61.67 ± 21.66 | 0.69 | 0.41 |
|  | Overhead concealment | 77.26 ± 1.45 | 67.00 ± 1.79 | 9.13 | 0.00 |
|  | Grass height | 74.82 ± 12.28 | 64.17 ± 23.97 | 0.42 | 0.52 |


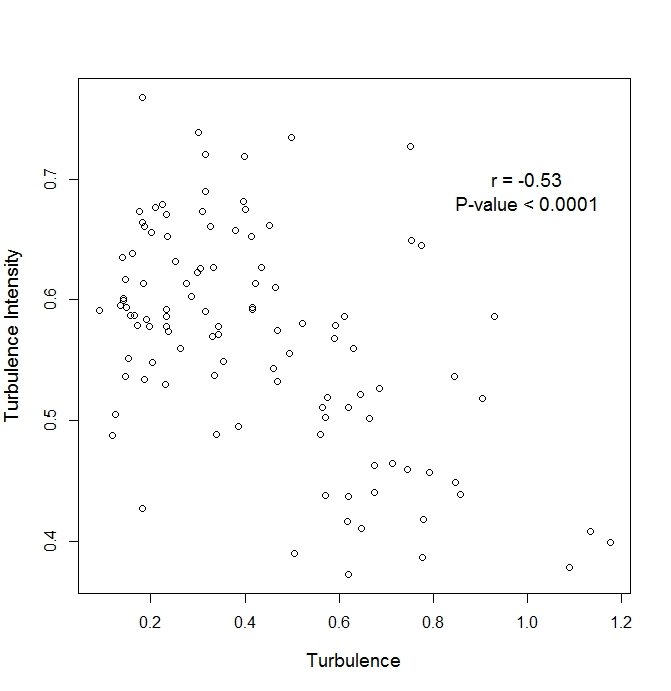
**Fig S1.** Turbulence intensity plotted against turbulence and the Pearson’s correlation coefficient (*r*) and P-value for this relationship in grasslands during 2015 and 2016 on the McFarlin-Ingersoll ranch, Inola, Oklahoma, USA.
